# The Impact of COVID-19 on Nursing Practice: Lessons Learned and Future Trends

**DOI:** 10.7759/cureus.50098

**Published:** 2023-12-07

**Authors:** Sirwan K Ahmed

**Affiliations:** 1 Department of Adult Nursing, College of Nursing, University of Raparin, Rania, IRQ

**Keywords:** mental health, artificial intelligence, nursing education, nursing practice, covid-19

## Abstract

The coronavirus disease 2019 (COVID-19) pandemic significantly impacted healthcare givers, especially nurses, who were at the forefront of the crisis. It highlighted the importance of preparedness, adaptability, and technology in healthcare systems. Nurses faced challenges like personal protective equipment (PPE) shortages, high patient numbers, and evolving protocols, emphasizing the need for robust plans and flexible infrastructures. Telehealth became crucial, ensuring care continuity and access, particularly for underserved groups. Mental health support for nurses became vital due to extreme stress and pressure. The pandemic also emphasized ongoing education and upskilling in nursing and highlighted the role of AI in healthcare solutions. Interdisciplinary collaboration among healthcare professionals gained importance, shaping a more holistic approach to patient care. This editorial highlights the impact of the COVID-19 pandemic on the nursing profession, highlighting the crucial significance of being well-prepared, adaptable, technologically advanced, and collaborative in order to ensure a more robust healthcare system in the future.

## Editorial

The coronavirus disease 2019 (COVID-19) pandemic stands as an unparalleled event that has significantly impacted various sectors of society, notably affecting healthcare professionals, particularly nurses. As frontline workers, nurses have borne the brunt of this crisis, encountering unparalleled challenges that have led to substantial alterations in the landscape of nursing practice [1]. This unparalleled situation has served as a pivotal juncture, catalyzing substantial change and prompting the emergence of innovative trends that are poised to significantly influence the future direction of nursing. Moreover, the pandemic has not only highlighted the immediate challenges but has also underscored the need for structural and systemic adaptations in healthcare delivery and nursing strategies, thereby necessitating comprehensive exploration and analysis for better understanding and preparedness in future healthcare crises. This editorial highlights the impact of the COVID-19 pandemic on the nursing profession, highlighting the crucial significance of being well-prepared, adaptable, technologically advanced, and collaborative in order to ensure a more robust healthcare system in the future.

The COVID-19 pandemic has underscored the indispensable significance of preparedness and adaptability within healthcare systems, particularly highlighting the essential role of nurses amidst the unprecedented challenges. Nurses were compelled to navigate unpredictable circumstances characterized by severe shortages of personal protective equipment (PPE), overwhelming patient caseloads, and the continual evolution of treatment protocols [2]. This crisis emphasized the urgent need for robust contingency plans and flexible healthcare infrastructures capable of rapid adjustment to unforeseen crises without compromising the quality of patient care or endangering the safety of healthcare staff. The global impact of COVID-19 has served as a clarion call for healthcare systems to reevaluate their preparedness strategies, advocating for resilience and adaptability as fundamental pillars in ensuring effective responses to future healthcare crises.

The COVID-19 pandemic brought forth an intensified recognition of the pivotal role technology plays in the domain of nursing. The adoption of telehealth and remote monitoring emerged as fundamental strategies enabling the delivery of healthcare while mitigating exposure risks [3]. Nurses exhibited rapid adaptation to diverse telemedicine platforms, proficiently conducting virtual consultations, remotely monitoring the vital signs of patients, and extending educational and supportive services. This transition toward telehealth not only ensured uninterrupted care provision amidst lockdown measures but also facilitated increased patient involvement and widened accessibility to healthcare services, notably benefiting marginalized and underserved communities. Additionally, the utilization of technology in nursing practice fostered innovative approaches, potentially reshaping healthcare delivery systems in a post-pandemic era.

Moreover, the COVID-19 pandemic significantly underscored the imperative need for robust mental health provisions and comprehensive support structures tailored for healthcare professionals, particularly nurses [4]. Throughout this global health crisis, nurses encountered unparalleled stressors due to the overwhelming nature of their responsibilities, bearing witness to widespread suffering and bereavement while enduring prolonged shifts amidst immense strain. This heightened the urgency for prioritizing mental health resources within healthcare institutions, necessitating tailored interventions to fortify resilience, mitigate burnout risks, and sustain the psychological well-being of nurses. To address these exigencies effectively, healthcare systems must implement multifaceted strategies encompassing accessible counseling services, peer support networks, and training programs aimed at equipping nurses with coping mechanisms and stress management skills. Establishing a resilient support framework is pivotal not only to alleviate immediate distress but also to fortify the long-term mental resilience of nursing professionals facing ongoing and future challenges in their practice.

Furthermore, the COVID-19 pandemic has prompted a critical reassessment of nursing education and training paradigms. The widespread transmission of the virus necessitated swift assimilation of new data and dynamic protocols, emphasizing the imperative for continuous educational advancement and proficiency enhancement among nursing professionals. This crisis underscored the crucial need to integrate comprehensive pandemic preparedness and crisis management modules within nursing curricula. This strategic integration aims to equip future nurses with a multifaceted skill set encompassing rapid adaptation, efficient crisis response, infection control measures, and the ability to deliver quality care amidst unprecedented healthcare challenges. Additionally, an emphasis on interprofessional collaboration, communication, and ethical considerations in pandemic scenarios has emerged as pivotal components of nursing education in preparing healthcare practitioners to navigate complex and evolving healthcare landscapes.

In the forthcoming landscape of nursing practice, several transformative trends are anticipated to influence the profession profoundly. Notably, the COVID-19 pandemic expedited the integration of artificial intelligence (AI) and data-centric healthcare approaches within nursing paradigms. Nurses are progressively embracing AI-driven instruments, utilizing them for predictive analytics, tailoring patient care to individual needs, and optimizing administrative functions, thus fortifying operational efficiency and augmenting patient outcomes [5]. This integration of AI in nursing encompasses a wide spectrum of applications, including but not limited to the analysis of vast healthcare datasets, facilitating diagnostic accuracy, and refining treatment strategies. Additionally, AI aids in the identification of potential health issues preemptively, allowing for proactive intervention and personalized care plans. Furthermore, AI-driven administrative tools assist in optimizing workflow efficiencies, thus enabling nurses to allocate more time toward direct patient care, enhancing overall quality and responsiveness within healthcare settings. The multifaceted adoption of AI in nursing practice not only enhances the precision and efficacy of healthcare delivery but also denotes a paradigm shift toward technology-enabled, patient-centric care approaches that are poised to define the future trajectory of the nursing profession.

The COVID-19 pandemic underscored the pivotal role of interdisciplinary collaboration within the healthcare milieu. Nurses, in particular, engaged in close collaboration with diverse healthcare stakeholders, including physicians, public health specialists, and various healthcare professionals, to develop and implement efficacious strategies and protocols for managing the pandemic's impact. This collaborative model is anticipated to persist, fostering an increasingly integrated and comprehensive approach to patient care. By leveraging the diverse strengths and expertise of each discipline involved, this collaborative framework not only optimizes patient outcomes but also enhances the overall effectiveness and efficiency of healthcare delivery. Furthermore, this approach extends beyond immediate crisis management, positioning interdisciplinary collaboration as an enduring cornerstone of healthcare, poised to shape future practices and promote a more cohesive, patient-centered healthcare landscape.

In conclusion, the COVID-19 pandemic has significantly impacted the field of nursing, representing a pivotal juncture that has instigated transformative changes and paved the way for future trends in nursing practice. It has unequivocally highlighted the indispensable requirement for comprehensive preparedness strategies within healthcare systems. Moreover, the pandemic underscored the pivotal role of technology integration, emphasizing the accelerated adoption of telehealth and remote monitoring tools in nursing care delivery. Simultaneously, it brought attention to the critical importance of addressing mental health challenges among healthcare professionals, particularly nurses, necessitating robust support systems and resources to mitigate burnout. Additionally, the crisis emphasized the necessity for continuous education and the upskilling of nurses to adapt to dynamic circumstances effectively. Notably, interdisciplinary collaboration emerged as a cornerstone in devising and implementing effective healthcare strategies during the pandemic. The ongoing adaptation and innovation among nurses in response to these challenges are poised to significantly influence and reshape the healthcare landscape, ensuring greater resilience, adaptability, and a more patient-centric approach in the foreseeable future. These lessons learned and evolving trends will serve as foundational elements in fostering a healthcare environment that is agile, responsive, and centered around optimizing patient care outcomes in the years ahead.
